# Social firms as a means of vocational recovery for people with mental illness: a UK survey

**DOI:** 10.1186/1472-6963-13-270

**Published:** 2013-07-11

**Authors:** Eleanor Gilbert, Steven Marwaha, Alyssa Milton, Sonia Johnson, Nicola Morant, Nicholas Parsons, Adrian Fisher, Swaran Singh, Di Cunliffe

**Affiliations:** 1 Caludon Centre, Coventry and Warwickshire Partnership Trust, Clifford Bridge Road, Coventry CV2 2TE, UK; 2 Division of Mental Health and Wellbeing, Warwick Medical School, University of Warwick, Coventry CV47AL, UK; 3 Early Intervention in Psychosis, Coventry and Warwickshire Partnership Trust, Swanswell Point, Stoney Stanton Road, Coventry, UK; 4 Department of Mental Health Sciences, University College London, 67-73 Riding House St, London W1W 7EJ, UK; 5 Department of Psychology, University of Cambridge, Downing Street, Cambridge CB2 3EB, UK; 6 Medical Statistician, Division of Health Sciences, Warwick Medical School, University of Warwick, Coventry CV47AL, UK; 7 Suresearch Office, Muirhead tower, University of Birmingham, Birmingham B152TT, UK; 8 Social Firms UK, Suite 2, Victoria House, 10 Brighton Road, Redhill RH1 6QZ, Surrey

**Keywords:** Employment, Recovery, Mental illness, Social firm, Social enterprise

## Abstract

**Background:**

Employment is associated with better quality of life and wellbeing in people with mental illness. Unemployment is associated with greater levels of psychological illness and is viewed as a core part of the social exclusion faced by people with mental illness. Social Firms offer paid employment to people with mental illness but are under-investigated in the UK. The aims of this phase of the Social Firms A Route to Recovery (SoFARR) project were to describe the availability and spread of Social Firms across the UK, to outline the range of opportunities Social Firms offer people with severe mental illness and to understand the extent to which they are employed within these firms.

**Method:**

A UK national survey of Social Firms, other social enterprises and supported businesses was completed to understand the extent to which they provide paid employment for the mentally ill. A study-specific questionnaire was developed. It covered two broad areas asking employers about the nature of the Social Firm itself and about the employees with mental illness working there.

**Results:**

We obtained returns from 76 Social Firms and social enterprises / supported businesses employing 692 people with mental illness. Forty per cent of Social Firms were in the south of England, 24% in the North and the Midlands, 18% in Scotland and 18% in Wales. Other social enterprises/supported businesses were similarly distributed. Trading activities were confined mainly to manufacturing, service industry, recycling, horticulture and catering. The number of employees with mental illness working in Social Firms and other social enterprises/supported businesses was small (median of 3 and 6.5 respectively). Over 50% employed people with schizophrenia or bipolar disorder, though the greatest proportion of employees with mental illness had depression or anxiety. Over two thirds of Social Firms liaised with mental health services and over a quarter received funding from the NHS or a mental health charity. Most workers with mental illness in Social Firms had been employed for over 2 years.

**Conclusions:**

Social Firms have significant potential to be a viable addition to Individual Placement and Support (IPS), supporting recovery orientated services for people with the full range of mental disorders. They are currently an underdeveloped sector in the UK.

## Background

Employment of people with mental health problems is associated with improved quality of life and wellbeing [1] and can also offer financial advantages both to individuals and society. Conversely unemployment is associated with greater levels of psychological illness in adults [2] and is viewed as a core part of the social exclusion faced by people with mental illness [3]. In the UK the rate of unemployment for people with severe mental illness is very high (80-90%) [4], though most individuals in this position state that they wish to work [5]. As well as the personal costs of unemployment the financial costs to the government are substantial, estimated to be £3.4 billion in 2005 for those with schizophrenia [6]. To address this issue, government policy in the UK has recommended that health and social care services should place greater focus on maintaining and promoting economic activity and vocational recovery for individuals with mental disorders [7].

Recovery as a concept in mental health services based around the idea that people with mental illness can build satisfying and hopeful lives [8], has rapidly gained prominence in recent years. It has become an important facet of mental health service provision in many countries [9-11], including the UK [12,13]. Employment has been described as a key factor providing a way of building a sense of meaning and purpose in life [14], and for those with mental health problems is now widely recognised as an important element in the multi-faceted recovery process. This has been repetitively reflected in recent UK government policy [15-17].

Obtaining a vocation has been found to have a positive impact on more life domains than almost any other medical or social intervention [18]. However, there remain multiple barriers to obtaining and maintaining employment for people with mental health problems. These include a lack of choice and opportunity, low levels of self esteem in relation to gaining work, coping with the pressures of working while managing symptoms of mental illness, stigmatization [19-22] and disincentives to employment that are embedded in the benefits system [22-24]. Additionally, the literature has highlighted the limited response of mental health services to meet the support needs of individuals to seek, gain and subsequently sustain employment [23,25]. To address this service gap vocational rehabilitation models have been developed, not only to facilitate a pathway to employment, but to also provide the individualised needs-based support needed to achieve this outcome [25].

There is a well documented shift toward vocational models that place less emphasis on pre-employment training experiences and a greater focus on real-world conditions and competitive jobs [26]. Current vocational schemes fall into three broad categories: social enterprises, traditional supported businesses, and supported employment [27]. Social enterprises are businesses with primarily social objectives. They generate at least half of their income from sales rather than government subsidies and their surpluses are principally reinvested in the business or community, rather than being distributed for individual profit. Social Firms, the main focus of the present paper, are a type of social enterprise where the social purpose is to provide employment to individuals who are disadvantaged in the labour market. In addition to the social enterprise guidelines, at least 25% of employees in a Social Firm face a major barrier to employment. Generally, Social Firm employees are paid at competitive rates. Managers are usually aware that their employees have previously been, or are currently impacted by social disadvantages such as mental illness. Such knowledge has the potential to improve the sustainability of the individual’s position at work [22]. The second type of vocational scheme is the traditional supported business model, previously known as sheltered employment. These models do not place parameters around their sources of income and thus can rely, sometimes heavily, on government subsidies. This model has reduced in popularity due to segregated settings, exclusively for individuals with disabilities [28] and the UK government has recently announced that it would be withdrawing subsidies to this type of scheme. The third and most established vocational pathway is supported employment, which aims to assist people to find employment in the open labour market. Individual placement and support (IPS) is a model of supported employment that places emphasis on client choice, rapid job finding, competitive jobs, integrated vocational settings, and follow-along support services [29].

IPS has a strong US evidence base [30], and reviews have found that supported employment models are more effective than prevocational training at helping people with severe mental illness obtain competitive employment [30-32]. Following reviews of the international research evidence, UK clinical guidelines from the National Institute for Health and Clinical Excellence have recommended supported employment, in particular the IPS approach, should be implemented for people with schizophrenia [33,34] and bipolar disorder [35]. Rinaldi and colleagues [36] have suggested that internationally there may be considerable cross-cultural differences between health, social care, employment and welfare systems. These factors may impact on success of establishing IPS in the UK, and thus require careful consideration upon implementation.

Presently, IPS studies incorporating UK samples are inconsistent [37,38] and a large number of service users in the UK do not engage with this model. Furthermore, the majority of those who do participate in these schemes do not obtain and sustain paid work. In 2009 Schneider and colleagues reported that only 25% of those motivated to work, obtained a paid job in routine settings through an IPS approach [39]. Under the IPS model there is also the potential for a proportion of individuals to continue to experience barriers to gaining and maintaining employment, such as stigma and the pressure of work whilst coping with mental illness. Social Firms, provide the potential opportunity to overcome some of these challenges as the ethos embedded in them advocates for a model of empowerment and recovery-oriented service [40,41]. For example, they have the potential to provide a stigma free environment where the employee’s mental health problems and the impact this has at work are accepted, with reasonable accommodations being incorporated in the workplace, organisational policies, and recruitment procedures.

Upon review of the current evidence base, there remains the possibility that a one size fits all vocational model would limit the opportunities for some individuals with mental health problems seeking employment. If there is to be a large and widespread change in employment patterns in this group in the UK, a variety of vocational pathways are likely to be required to meet a range of needs. Social Firms have the potential to complement the IPS model by being part of a range of services that can promote social inclusion of the mentally ill in employment as well as presenting a prosperous business model [42,43]. This may be of particular benefit for service users who do not succeed in obtaining work through the IPS support structure or others who decide it is not suited to their situation.

Policy endorsements of vocational pathways for individuals with mental health issues have coincided with an increasing number of Social Firms across the UK. Before 1997 there were just six Social Firms in Britain; by 2006, the number had grown to 49, plus 70 “emerging” Social Firms [44]. Like many other small enterprises, the recent recession has led to a downturn in these businesses in the UK (personal communication - Social Firms UK 2012), and when compared to the rest of Europe, Social Firms remain considerably less common [44]. From a research perspective, Social Firms have undergone limited evaluation in the UK [18]. A small qualitative study from Scotland reported that employment in Social Firms has postive impacts on individuals’ social connection and feelings of personal competence [41]. Although this shows promise for both the vocational and non-vocational outcomes for employees, there is an insufficient level of research on Social Firms in the UK to infer that they represent a viable health and social care policy strategy.

If Social Firms are a viable alternative employment pathway model in the UK and are to be supported in mental health policy, there is a need for preliminary research to strengthen the currently limited knowledge base. Thus in this phase of the Social Firms A Route to Recovery (SoFARR) project we aimed to describe the availability and spread of Social Firms across the UK, provide an outline of the range of opportunities and support Social Firms offer, and understand the extent to which people with severe mental illness are employed within these firms.

## Methods

The study was given a favourable ethical opinion by the National Research Ethics Committee West Midlands.

A UK national survey was completed between March and July 2011 of Social Firms and other types of social enterprise or supported business that provide paid employment for people with mental illness. Businesses that provided only work experience or vocational training were not included. Membership lists of Social Firms UK, Social Enterprise UK, Social Traders, British Association for Supported Employment and The International Centre for Clubhouse Development were searched. A snowballing technique was adopted whereby those contacted were asked if they could advise about other relevant businesses. All UK local authorities were contacted along with 21 Job Centre Plus offices. Lead occupational therapists in all Mental Health Trusts in England and Wales and Health Boards in Scotland were approached and asked to provide details of any relevant businesses they were aware of. National mental health charities and academics with an interest in mental health and employment were also contacted. An internet search was carried out in order to identify any additional businesses specifically providing paid employment for people with mental illness.

From our initial search it was clear that the number of Social Firms currently employing people with mental health problems was lower than had been anticipated. For this reason other types of social enterprise and supported businesses identified in the search were included in the survey. This provided an opportunity to conduct basic comparisons between the different employment settings in terms of the diagnoses, job roles, rates of pay etc. of those individuals with mental health problems who were employed there and the financial circumstances of the businesses. It was not our intention to carry out a detailed exploration or evaluation of the different employment support models.

A study-specific questionnaire (Additional file 1) was developed to determine how Social Firms operate in providing employment to people with mental illness and piloted with Social Firm managers. A slightly modified version of the questionnaire was sent to businesses that were not registered as Social Firms (e.g. supported businesses) to ensure that terminology matched the business status. The questionnaire covered two broad areas, asking the employers about the nature of the Social Firm itself and then also about the employees with mental illness working there. Questions about the Social Firm included the length of time operating as a Social Firm, goods or services supplied, payment of employees, business finances and turnover, sickness management, recruitment of workers with mental illness and liaison with mental health services. Information requested about employees was number with a history of mental illness, diagnosis, medication and time off work for mental health appointments or illness.

Questionnaires were sent to businesses by post, with an assurance that all information provided would be treated as confidential and that no identifying information would be disclosed in any publications; non-returns were followed up by telephone and email correspondence. A total of 67 Social Firms (all identified from Social Firms UK membership list) and 82 social enterprises/supported businesses were identified from our initial search as potential employers of people with a history of mental health problems. The majority of the social enterprises/supported businesses (69) were identified through membership lists of the relevant bodies. A further 5 were identified through approaches to Mental Health Trusts, 5 through snowballing and 3 by academics with an interest in this area.

## Results

Responses were obtained from 145 (97%) employers with 86 confirming that they currently employed at least one person with mental illness. Ten businesses that confirmed that they did employ people with a history of mental illness provided only information on the numbers employed and goods or service provided, but did not complete the full questionnaire. Complete (or substantially completed) questionnaire returns were received for 88% (n = 76) of the eligible businesses representing 692 employees with mental illness. One questionnaire was returned from a head office of a national supported business that provided amalgamated data for 24 supported businesses that had been sent individual questionnaires, and so for the purpose of analysis this has been treated as one response. Results are therefore based on questionnaire data from 33 Social Firms and 20 other social enterprises or supported businesses. Results for businesses are shown in Table 1 and for employees in Table 2.

**Table 1:** Businesses employing people with mental illness

	**Social firms (n = 33)**		**Social enterprise or supported business (n = 20)**	
Location^†^				
*England, South*	15	(39.5%)	15	(60%)
*England North and Midlands*	9	(23.7%)	6	(24%)
*Scotland*	7	(18.4%)	4	(16%)
*Wales*	7	(18.4%)	0	(0%)
Trade^†^				
*Manufacturing/packing/printing*	11	(28.9%)	15	(60%)
*Service industry*	9	(23.7%)	4	(16%)
*Recycling/horticulture*	11	(28.9%)	3	(12%)
*Catering*	7	(18.4%)	3	(12%)
Mean number of years operating(range)	6.8(n = 32)	(2–26)	24(n = 19)	(1–65)
Annual turnover (mean; k = £1,000)	£460 k(n = 25)	(£38 k-£2000 k)	£819 k(n = 13)	(£20 k-£4500 k)
Mean percentage of income from goods/services	83%(n = 20)		70%(n = 16)	
Business makes a profit each year	17(n = 25)	68%	6(n = 18)	33%
Mean number of employees (range)	15.2(n = 21)	(2–56)	36.5(n = 12)	(5–130)
Median number of employees with MH problems (range) ^†^	3(n = 32)	(1–70)	6.5(n = 24)	(1–110)
Business has MH employees in management	13(n = 26)	50%	6(n = 16)	37.5%
Businesses employing people with				
*Schizophrenia*	11(n = 22)	(50%)	7(n = 13)	(53.8%)
*Bipolar Disorder*	6(n = 21)	(28%)	7(n = 12)	(58.3%)
*Anxiety / Depression*	15(n = 21)	(71.4%)	11(n = 12)	(91.6%)
MH sickness impacts on day to day running of business	13(n = 31)	(41.9%)	12(n = 20)	(60%)
MH sickness impacts on long term viability of business	5(n = 31)	(16.1%)	7(n = 20)	(35%)
Receives income from NHS or mental health charity	10(n = 32)	(31.2%)	3(n = 20)	(15%)
Business liaises with mental health service	22(n = 32)	(68.7%)	14(n = 20)	(70%)

† n = 38 Social Firms, n = 25 supported businesses/social enterprises as includes basic information from firms who did not complete questionnaire.

**Table 2:** Employees with mental illness

	**Social firms**			**Social enterprise**/**Supported business**		
	**Employees**		**Businesses**	**Employees**		**Businesses**
Period of time employee in post (%)						
*< 6 months*	14	(11.9%)	27	20	(4.9%)	18
*6 months – 1 year*	22	(18.8%)	27	29	(7.2%)	18
*1 – 2 years*	20	(17.0%)	27	15	(3.7%)	18
*> 2 years*	61	(52.1%)	26	340	(84.1%)	18
Rates of pay (%)						
*Minimum wage*	25	(21.9%)	28	45	(13.2%)	18
*Minimum wage + up to £3.00*	36	(31.6%)	27	231	(74.3%)	18
*Minimum wage + up to £5.00*	35	(30.7%)	27	26	(9.3%)	18
*Other amount*	16	(14.0%)	27	5	(1.6%)	18
Proportion of employees with MHP						
*Schizophrenia*	15	(14.1%)	22	33	(24.2%)	13
*Bipolar Disorder*	11	(10.4%)	22	14	(10.3%)	13
*Anxiety / Depression*	46	(43.4%)	22	56	(41.2%)	13
*Other/not known*	72	(32.0%)	22	33	(24.2%)	13
Employee takes regular time off for mental health appointments	36	(33.9%)	23	34	(22.6%)	13
Employee takes regular mental health medication	53	(50.0%)	17	94	(55.2%)	14
Mean MH employees days per year sick	8.4		23	14.5		10
Mean other employees days per year sick	5.3		21	32.5		14

The Social Firms surveyed were located throughout the UK. Forty per cent were in the South of England, 24% in the North and the Midlands, 18% in Scotland and 18% in Wales. Other social enterprises/supported businesses were similarly distributed, with 60% in the South of England, 24% in the North and Midlands and 16% in Scotland. There were no responses received from other social enterprises/supported businesses in Wales. The scope of trading activities was confined mainly to manufacturing, service industry, recycling, horticulture and catering. Thirty two per cent of Social Firms included people with other disabilities in their target groups in addition to people with mental health problems; this information was not available for supported businesses/social enterprises.

The mean number of years of operation was 6.8 for Social Firms and 24 for social enterprises/supported businesses. The numbers of employees with mental illness working in the Social Firms and other social enterprises/supported businesses was small with a median of 3 and 6.5 respectively. The median number of employees in total was 10 (n = 341) in Social Firms and 25 (n = 2738) in supported businesses/social enterprises. Seventeen Social Firms and 13 supported businesses/social enterprises did not provide data about total numbers of employees. Half of Social Firms reported that an employee with mental health problems held a management position in the business compared to 37.5% of supported businesses/social enterprises. Fifty two per cent of employees with a history of mental illness working in the Social Firms had been there for longer than two years. In social enterprises and supported businesses this figure rose to 84.1%. The majority of employees of both types of business were paid at above the UK minimum wage, with 30% of employees of Social Firms being paid at UK minimum wage plus up to £5.00 per hour compared to 9.3% of those employed in supported businesses/social enterprises. A greater proportion of Social Firms reported making a profit each year than social enterprises/supported businesses (68% and 33% respectively) and more of their income was generated through the sale of goods or services (83.4% and 70% respectively).

Both Social Firms and social enterprises/supported businesses employed people with a range of mental health problems. Fifty per cent of Social Firms and 53.8% of social enterprises/supported businesses employed people with schizophrenia. The proportion of businesses employing people with a diagnosis of bipolar disorder was lower than this for Social Firms (28%) but higher for social enterprises/supported businesses (58.3%). The majority of firms employed people with depression or anxiety (71.4% of Social Firms, 91.6% of other businesses) and in both Social Firms and social enterprises/supported businesses the greatest proportion of employees with mental illness had depression or anxiety (43.4% and 41.2% respectively). Employees with schizophrenia made up the next largest proportion (14.1% for Social Firms, 24.2% for social enterprises/supported businesses). Both types of business employed similar proportions of people with bipolar disorder at around 10%.

Forty two per cent of Social Firms and 60% of social enterprises/supported businesses reported that sickness absence among employees with mental illness impacted on the day to day running of the business. However, a smaller proportion of both types of business reported that sickness levels of employees with mental health problems had an impact on the long term viability of the business (16.1% and 35% respectively). At least half of employees with mental health problems in both types of business took regular psychotropic medication. A third of these Social Firm employees took time off work for mental health appointments, as did just under a quarter of social enterprises/supported business employees with mental health problems. Workers with a history of mental illness in Social Firms in comparison to those without took a slightly higher number of sick days a year (8.4 and 5.3 respectively). This was lower than for those with and without mental illness who were working in social enterprises/supported businesses, who took an average of 14.5 and 32.5 days per year respectively. Over two thirds of Social Firms and other businesses liaised with mental health services. Social Firms were more likely to receive funding from the NHS or a mental health charity than social enterprises/supported businesses (31.2% of Social Firms, 15% of social enterprises/supported businesses).

## Discussion

Employment of people with a history of mental illness in Social Firms is currently on a very small scale in the UK. Although the Social Firms sector is fairly new to the UK it grew by 32% between 2006 and 2010 [45]. The Social Firms participating in this study tended to have been developed in the last decade, whereas the supported businesses had mainly been in existence for much longer, sometimes for over 60 years. Although the majority of Social Firms have very small numbers of employees with mental illness, half of them had people with mental illness working in management positions. As in supported businesses and other social enterprises, the majority of people with mental illness working in Social Firms had been in their paid posts for longer than two years, suggesting that the jobs were relatively stable and sustainable. This compares well with mean job tenure of 17.5 weeks at 18 month follow-up reported in a recent meta-analysis of four IPS programmes [46].

A greater proportion of employees with mental health problems within Social Firms have rates of pay of up to £5.00 above the minimum UK wage than those working in supported businesses or social enterprises. However, more Social Firms made a profit each year than supported businesses and social enterprises and a greater proportion of their income was generated through the sale of goods and services, suggesting that Social Firms therefore may not be subject to the criticisms directed at sheltered work schemes that work is unpaid or underpaid and outside the needs of a viable commercial business [47].

Despite the expansion of the Social Firms sector over recent years and the fact that people with mental health problems are the largest target group [45] the actual numbers of employees with mental health problems in the sector remains low (245 in our survey), and is a tiny proportion of the number of people in the UK with severe mental illness. Of the Social Firms who target people with mental health problems over a quarter did not currently employ anyone in this group. This may reflect the fact that many Socials Firms have multiple target groups (12 of the Social Firms in our survey also included people with other disabilities in their target group) and are small, employing few people overall. Half of those in our survey employed less than 10 people in total. Combined with the slow turnover of employees with mental illness it is not surprising therefore that opportunities for people with mental health problems to find employment in a Social Firm are limited at present.

The sickness absence rate of around 8 days per year for Social Firm employees with a mental illness is higher than the UK average of 4.5 days [48], but not dramatically so, perhaps reflecting workers views that employment in a Social Firm aids recovery from mental illness [41], a common theme expressed by patients about a return to work [22]. These figures would also suggest that the unease of many employers about the capabilities of mentally ill workers and concerns about frequency of absence [49] are largely unfounded. Survey results found that 42% and 60% of managers of Social Firms and social enterprises/supported businesses respectively reported that the sickness levels of mentally ill employees affected the day to day running of the business. However, fewer Social Firm managers saw this as potentially influencing the long term viability of the business, suggesting that there are strategies in place to ameliorate the impact of the higher sickness rate among employees with mental health problems.

Over 50% of Social Firms and other businesses surveyed have employees with mental illnesses that are routinely seen in secondary care such as schizophrenia and bipolar disorder. It does not therefore seem that Social Firms only offer paid work to those with common mental illness such as anxiety or depression. This finding was consistent with previous anecdotal evidence that Social Firms can accommodate people who have been admitted to psychiatric hospital [50].

The majority of Social Firm managers were aware of the diagnosis of their employees with mental health problems and whether they were taking medication for their condition. More than two thirds of the Social Firms surveyed said they liaised with their local mental health services, either to recruit workers or in order to support employees with their difficulties. This level of support and communication with mental health services might allay patient, carer and clinician’s concerns that work might be harmful to patient recovery [51]. Furthermore this may open up a collaborative pathway that assists mental health services shift further towards recovery-oriented practices including helping people to work.

Under supported business and IPS models of supported employment there remains the potential for a proportion of individuals to continue to experience barriers to gaining and maintaining employment, such as stigma and the pressure of work whilst coping with mental illness. Social firms provide the potential opportunity to overcome some of these challenges as the ethos embedded in them advocates for a model of empowerment and recovery-oriented service [40,41]. For example, they have the potential to provide a stigma free environment where the employee’s mental health problems and the impact this has at work are accepted; with reasonable accommodations being incorporated in the workplace, the organisational policies, and recruitment procedures [52]. Greater opportunities provided by Social Firms in comparison to social enterprises/businesses for people with mental illness to develop and progress to management positions within the organisation may promote recovery through continuity, improved self esteem and purpose.

### Research/policy implications

Exploiting and expanding channels of communication between Social Firms and the NHS seems a potentially fruitful way to support the employment of people with a range of mental health conditions, and would be in line with UK government recommendations that health and social care services should place a greater focus on maintaining and promoting vocational recovery for individuals with mental illness. However, the underlying mechanisms that assist these individuals to engage in Social Firm employment, what they gain from this type of employment, and whether the Social Firms model of vocational support varies across different workplaces has not been explored. Understanding these aspects of the Social Firm model from the perspective of both those with mental health problems and managers of Social Firms may assist to inform future development of the sector and expansion of employment initiatives across the UK. A clearer understanding of the relative benefits of Social Firms in comparison to other social enterprises/businesses in promoting recovery is also needed.

### Limitations

Despite a rigorous search strategy it is possible that some Social Firms or other social enterprises/supported businesses employing people with mental illness were missed in the survey or have been established since the survey was conducted. Some businesses were reluctant to disclose financial information on the grounds that it was commercially sensitive, and for this reason financial results are based on a smaller number of firms. Information about the diagnosis and management of individual employees’ mental health was not known by around a third of managers, so again the results relating to diagnosis and management of mental ill health are based on information from fewer firms.

## Conclusions

Our research thus far has established a macro level understanding of Social Firms opportunities for employees with mental health problems. Although the number of Social Firms has grown in recent years they are still a very underdeveloped sector currently in the UK. Social Firms could represent a viable addition to IPS and appear to provide opportunities to gain sustained employment, supporting personal and vocational recovery for people with the full range of mental disorders. However further research using symptomatic and disability measures and in-depth interviews with employees, Social Firm managers and clinicians is required to gain understanding of the characteristics of people who are working in a Social Firm and the reasons they choose to do so. This will form the focus of our further study in this programme of research.

## Competing interests

Di Cunliffe is an employee of Social Firms UK, an umbrella organisation which promotes Social Firms in the UK.

## Authors’ contributions

EG conducted the questionnaire survey and analysis of the data. EG, AM and SM initially drafted the manuscript. All authors contributed to the interpretation of the data. SM and SJ conceived of the study and its design. All authors have been involved in revising the manuscript and all authors read and approved the final manuscript.

## Funding

This research was funded through the National Institute of Health Research: Research for Patient Benefit Programme. This report represents independent research commissioned by the National Institute for Health Research. The views expressed in this publication are those of the author(s) and not necessarily those of the NHS, the National Institute for Health Research or the Department of Health.

## Pre-publication history

The pre-publication history for this paper can be accessed here:

http://www.biomedcentral.com/1472-6963/13/270/prepub

## Supplementary Material

Additional file 1 Questionnaire sent to Social Firms. Click here for file
