# Supplementary material for: Social firms as a means of vocational recovery for people with mental illness: a UK survey
Source: BMC Health Serv Res. 2013 Jul 11;13:270. doi: 10.1186/1472-6963-13-270 (PMC3710483; doi:10.1186/1472-6963-13-270)
Supplement: Additional file 1 — Questionnaire sent to Social Firms. [file 1472-6963-13-270-S1.doc]

:

**SoFARR - (Social Firms a Recovery Route)**

***Thank you for taking the time to complete this survey. We have tried to keep it as brief as possible and expect that it will take no longer than 20 minutes to complete. If you are unsure of exact figures, please provide your best estimate.***

**About your social firm**

1. **Name of firm:**

**2. Address:**

**3. How long has the firm been a social firm?**

**4. How long has the firm been operating as a business?**

**5. What is the legal status of your firm?**

| Company limited by guarantee or shares |  |
| --- | --- |
| Registered charity |  |
| Subsidiary of charity/other parent organisation |  |
| Statutory organisation |  |
| Cooperative |  |
| Other (please state) |  |

**6. What services or goods do you sell?**

**About your workforce:**

1. **Are trainees with mental health problems paid?**

Yes No Don’t know

1. **How much are employees/trainees who have mental health problems paid per hour?**

| **Hourly wage** | **Number of *employees* with**  **mental health problems paid in this range** | **Number of *trainees* with mental health problems paid in this range** |
| --- | --- | --- |
| Less than minimum wage* |  |  |
| Minimum wage |  |  |
| Minimum wage + up to £3.00 |  |  |
| Minimum wage + £3.01 to £5.00 |  |  |
| Other (please state) |  |  |

*** £5.93 age 21 and over; £4.92 age 18-20; £3.64 age 16-17**

1. **How many of your employees who have mental**

Don’t know

**health problems hold management positions in**

**the firm?**

1. **How many employees who have a history of mental**

Don’t know

**health problems have been promoted in the last 5 years?**

**Sickness absence:**

1. **On average, how many days per year do your**

Don’t know

**employees *with* mental health problems take as**

**sick leave?**

1. **On average, how many days per year do your**

Don’t know

**employees *without* mental health problems take**

**as sick leave?**

1. **How many of your employees with mental health**

**problems have the following diagnoses?**

| **Diagnosis** | **Number** | **Don’t know** |
| --- | --- | --- |
| **Schizophrenia /psychosis** |  |  |
| **Manic depression** |  |  |
| **Depression and anxiety** |  |  |
| **Substance misuse** |  |  |

1. **How many of your employees have regular time off**

Don’t know

**to attend appointments with a psychiatrist, CPN or**

**counsellor?**

Don’t know

1. **How many of your employees take regular medication**

**for their mental health problem?**

1. **Does the firm have set procedures for managing**

Yes No Don’t know

**sickness absence?**

**17. Is the procedure less strict for employees with**

Yes No Don’t know

**mental health problems?**

1. **Do sickness absence rates for employees**

**with mental health problems have an impact on:**

**i) Day to day running of the firm?**

Yes No Don’t know

**ii) Long-term viability of the firm?**

Yes No Don’t know

1. **For how many days are employees able to self-certify sickness?**

Yes No Don’t know

**20. Does the firm liaise with any mental health services?**

**21. If yes, which services?**

**22. What is the purpose of the liaison?**

**Recruitment and turnover:**

1. **Do employees with a history of mental health**

Yes No Don’t know

**problems usually work as volunteers or trainees**

**within the firm before being fully employed by you?**

1. **Where do you advertise vacancies within your firm?**
2. **How long have your current employees been working for the firm?**

| **Length of service** | **Number of employees with mental health problems** | **Number of employees without mental health problems** |
| --- | --- | --- |
| Less than 6 months |  |  |
| 6 months to a year |  |  |
| 1 to 2 years |  |  |
| More than 2 years |  |  |

1. **How many employees who have a history of mental health problems** have left the firm for the following reasons in the last 5 years?

| **Reason for leaving the firm** | **Number of employees with mental health problems** |
| --- | --- |
| Work in another social firm? |  |
| Work in non-social firm |  |
| Sheltered/supported work |  |
| Voluntary work |  |
| Training (mainstream) |  |
| Training (supported) |  |
| Unemployment |  |
| Illness—need acute mental health services |  |
| Other (please state) |  |

**Finance**

1. **Does your firm receive any income outside of**

Yes No Don’t know

**the sale of goods or services?**

1. **If yes, where from?**
2. **If you are not registered with Social Firms UK, what**

% Don’t know Not applicable

**proportion of your income is from the sale of goods**

**and services?**

1. **Does any of your income come from an**

Yes No Don’t know

**NHS Mental Health Trust/ mental health**

**charity or other mental health body?**

1. **What is the annual turnover of your business?**

Yes No Don’t know

1. **Does your business make a profit each year?**
2. **If yes, how is the profit used?**

**34. Are there any other comments you would like to make about employing people with mental health problems within your Social Firm or Social Enterprise?**

1. **If you are aware of any other local businesses operating as Social Firms or Social Enterprises employing people with a history of mental health problems, please provide details below. This will help to ensure that we contact as many relevant employers as possible.**

**Thank you very much for completing this survey.**
